# The Brain Functional Networks Associated to Human and Animal Suffering Differ among Omnivores, Vegetarians and Vegans

**DOI:** 10.1371/journal.pone.0010847

**Published:** 2010-05-26

**Authors:** Massimo Filippi, Gianna Riccitelli, Andrea Falini, Francesco Di Salle, Patrik Vuilleumier, Giancarlo Comi, Maria A. Rocca

**Affiliations:** 1 Neuroimaging Research Unit, Institute of Experimental Neurology, Division of Neuroscience, Scientific Institute and University Hospital San Raffaele, Milan, Italy; 2 Department of Neurology, Scientific Institute and University Hospital San Raffaele, Milan, Italy; 3 Department of Neuroradiology, Scientific Institute and University Hospital San Raffaele, Milan, Italy; 4 Maastricht Brain Imaging Center, Department of Cognitive Neuroscience, University of Maastricht, Maastricht, The Netherlands; 5 University Medical Center of Geneva, University of Geneva, Geneva, Switzerland; Cuban Neuroscience Center, Cuba

## Abstract

Empathy and affective appraisals for conspecifics are among the hallmarks of social interaction. Using functional MRI, we hypothesized that vegetarians and vegans, who made their feeding choice for ethical reasons, might show brain responses to conditions of suffering involving humans or animals different from omnivores. We recruited 20 omnivore subjects, 19 vegetarians, and 21 vegans. The groups were matched for sex and age. Brain activation was investigated using fMRI and an event-related design during observation of negative affective pictures of human beings and animals (showing mutilations, murdered people, human/animal threat, tortures, wounds, etc.). Participants saw negative-valence scenes related to humans and animals, alternating with natural landscapes. During human negative valence scenes, compared with omnivores, vegetarians and vegans had an increased recruitment of the anterior cingulate cortex (ACC) and inferior frontal gyrus (IFG). More critically, during animal negative valence scenes, they had decreased amygdala activation and increased activation of the lingual gyri, the left cuneus, the posterior cingulate cortex and several areas mainly located in the frontal lobes, including the ACC, the IFG and the middle frontal gyrus. Nonetheless, also substantial differences between vegetarians and vegans have been found responding to negative scenes. Vegetarians showed a selective recruitment of the right inferior parietal lobule during human negative scenes, and a prevailing activation of the ACC during animal negative scenes. Conversely, during animal negative scenes an increased activation of the inferior prefrontal cortex was observed in vegans. These results suggest that empathy toward non conspecifics has different neural representation among individuals with different feeding habits, perhaps reflecting different motivational factors and beliefs.

## Introduction

Social cognition includes mental processes necessary to understand and store information about the self and other persons, as well as interpersonal norms and procedures to navigate efficiently in the social world [1]. Basic abilities underlying social cognition include the perception and evaluation of social stimuli, the integration of perceptions with contextual knowledge, and finally the representation of possible responses to the situation. One of the hallmarks of social cognition in humans is the ability to understand conspecifics as beings like oneself, with intentional and mental lives like one's own [2]. Accordingly, human beings tend to identify with conspecifics and attribute mental states to them. Such abilities rely on the activity of several brain regions, including the frontal lobes (orbitofrontal cortex, medial prefrontal cortex, and cingulate cortex), the temporal lobes (including the amygdala), the fusiform gyrus, and the somatosensory cortices [3], [4], [5]. The majority of these regions is also critically involved in the processing of emotions [6]. This suggests that the merging between emotions and feelings experienced by oneself and those perceived in other individuals may be a key ingredient of social understanding, and it may play a major role in promoting empathy, prosocial behaviours, and moral norms [1], [3]. Moreover, empathic responses can be modulated by the subjective attitude held toward suffering individuals [7], as well as by personal experience [8]. Several functional magnetic resonance imaging (fMRI) studies showed that observing the emotional state of another individual activates a neuronal network involved in processing the same state in oneself, whether it is pain, disgust, or touch[3], [4], [5]. Empathy toward another person, which can be defined as the ability to share the other person's feeling in an embodied manner, has been related to recruitment of a network mostly including the somatosensory and insular cortices, limbic regions and the anterior cingulate cortex (ACC). Whereas cognitively inferring about the state of other person (known as theory of mind) has been associated with recruitment of medial prefrontal regions, the superior temporal sulcus and the temporo-parietal junction[4].

A few investigations have also assessed whether affective links between people modulate their brain empathic responses to others, such as when these are loved ones or strangers[9], or when they are believed to be fair or unfair persons [7], [9]. The majority of previous studies attempting to characterize empathy-related responses did not separate empathy towards humans from that towards animals. Furthermore, in some studies, scenes showing animals were treated as a neutral condition. However, a recent study [10] that compared stimuli depicting human and non human animal targets demonstrated higher subjective empathy as the stimuli became closer in phylogenetic relatedness to humans (mammalian *vs*. bird stimuli), thus indicating that empathic response towards humans may generalize to other species.

In this study, we postulated that the neural representation of conditions of abuse and suffering might be different among subjects who made different feeding choice due to ethical reasons, and thus result in the engagement of different components of the brain networks associated with empathy and social cognition. In details, we tested the hypothesis that the neural processes underlying empathy in vegetarians and vegans may not only operate for representations about humans but also animals, and thus vary between them and omnivore subjects. Vegetarians and vegans, who decided to avoid the use of animal products for ethical reasons, have a moral philosophy of life based on a set of basic values and attitudes toward life, nature, and society, that extends well beyond food choice. The earliest records of vegetarianism as a concept and practice among a significant number of people was closely connected with the idea of nonviolence towards animals and was promoted by religious groups and philosophers. The term veganism, which was coined from vegetarianism, acknowledges the intrinsic legitimacy of all sentient life and rejects any hierarchy of acceptable suffering among creatures. Veganism is a lifestyle that seeks to exclude the use of animals for food, clothing, or any other purpose [11]. The central ethical question related to veganism is whether it is right for humans to use and kill animals. Due to these differences of believes and behaviours, we also hypothesized that, in addition to a common shared pattern of cortical processing of human and animal suffering, vegetarians and vegans might also have functional architecture differences reflecting their different motivational factors and believes.

## Results

### Empathy assessment

**Figure 1:**
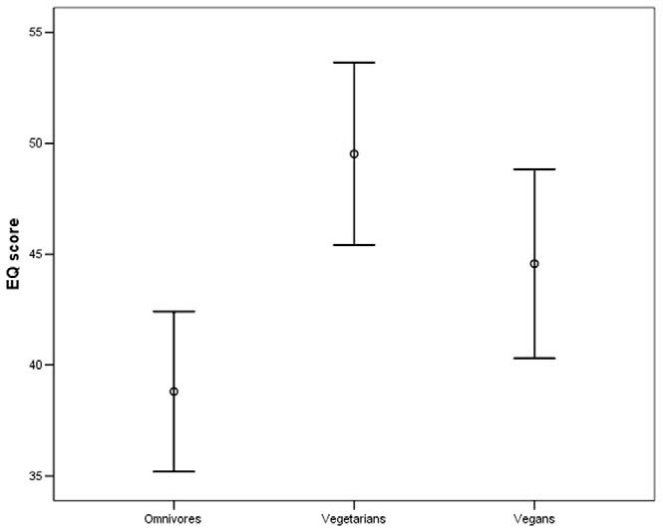
Graph showing error bars of means and standard deviations of empathy quotient (EQ) score in the three groups of subjects. See text for further details.

The Empathy quotient (EQ) score was significantly different between groups (p = 0.002). At post-hoc analysis, the EQ score was significantly higher in vegetarians in comparison with omnivore subjects (mean EQ score = 49.5, SD = 8.9 in vegetarians *vs*. 38.8, SD = 8.1 in omnivore; p = 0.001), and in vegans (mean EQ score = 44.6, SD = 9.8) in comparison with omnivore subjects (p = 0.04) (Figure 1). The difference between vegans and vegetarians was not statistically significant.

### Within-group fMRI results

**Figure 2:**
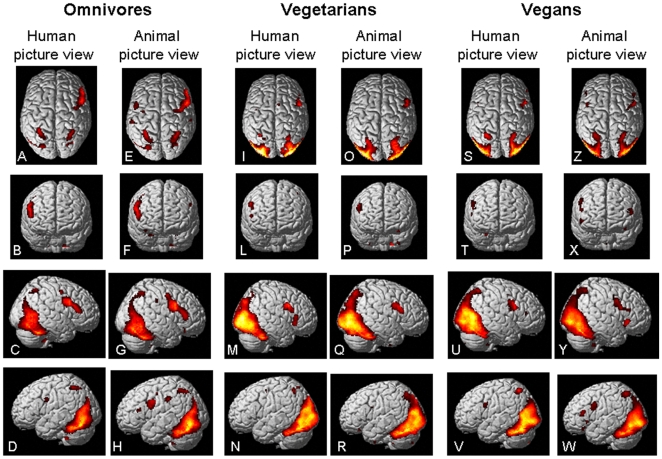
Within-group analysis of activations. Cortical activations on a rendered brain from omnivore (A–H), vegetarian (I–R) and vegan (S–W) subjects during observation of pictures showing negative valence scenes of humans (A–D, I–N, S–V) or animals (E–H, O–R, Z–W) (within-group analysis, one-sample t tests, t = 3 for display purpose). Images are in neurological convention.

The observation of both human and animal negative valence scenes resulted in the recruitment of several brain areas involved in emotion and empathy in the three groups of subjects, including the anterior insula, basal ganglia, thalami, and several other cortical areas located in the occipital lobes, prefrontal and parietal cortices. Figure 2 shows the brain patterns of activations in the three groups of subjects during the different experimental conditions. Table 1 summarizes the main results of within-group comparisons of the two experimental conditions.

**Table 1:** Within-group comparisons of human *vs*. animal negative valence picture view and vice versa in omnivore subjects, vegetarians and vegans (paired t test in each group, p<0.05 FWE-corrected).

		Human *vs*. animal pictures			Animal *vs*. human pictures		
		Omnivore	Vegetarians	Vegans	Omnivore	Vegetarians	Vegans
Activation sites	BA	MNI coordinatesX Y Z	MNI coordinatesX Y Z	MNI coordinatesX Y Z	MNI coordinatesX Y Z	MNI coordinatesX Y Z	MNI coordinatesX Y Z
R amygdala	-	-	30, −16, −22	26, −8, −18	22, −4, −26	-	-
R MTG	37	46, −64, 2	60, −32, 2	44, −64, 1056, 8, −28	-	-	-
L MTG	37	−48, −74, −6	-	−62, −50, 2−52, −2, −22	-	-	-
R lingual gyrus	19	14, −54, −2	-	-	-	-	-
L lingual gyrus	19	−10, −66, −2	-	-	-	-	-
R cuneus	18	16, −80, 28	14, −86, 28	-	-	-	-
R precuneus	7	8, −54, 43	-	-	-	-	-
R insula	-	-	38, 24, 8	-	-	-	-
R thalamus	-	-	-	20, −26, 0	-	-	-
R putamen	-	-	-	30, −6, −4	-	-	-
L putamen	-	-	-	−28, 6, −10	-	-	-
R IOG	-	-	-	-	20, −86, −12	-	-
L IOG	-	-	-	-	−30, −82, −10	-	−50, −66, −14
L IPL	40	-	-	-	−48, −56, 46	-	-
R MFG	-	-	-	-	38, 4, 50	-	-
L MFG	-	-	-	-	−40, 14, 36	-	-
R IFG	-	-	-	-	44, 44, −8	-	46, 22, −8
ACC	32	-	-	-	-	0, 50, 10	-
PCC	23	-	-	-	-	2, −50, 20	0, −40, 26

MNI = Montreal Neurological Institute, R = right, L = left, BA = Brodmann area, MTG = middle temporal gyrus, IOG = inferior occipital gyrus, IPL = inferior parietal lobule, MFG = middle frontal gyrus, IFG = inferior frontal gyrus, ACC = anterior cingulate cortex, PCC = posterior cingulate cortex.

### Between-group fMRI results

The patterns of activations during the neutral condition did not differ between groups.

### Common regions of activations between vegetarians and vegans

**Figure 3:**
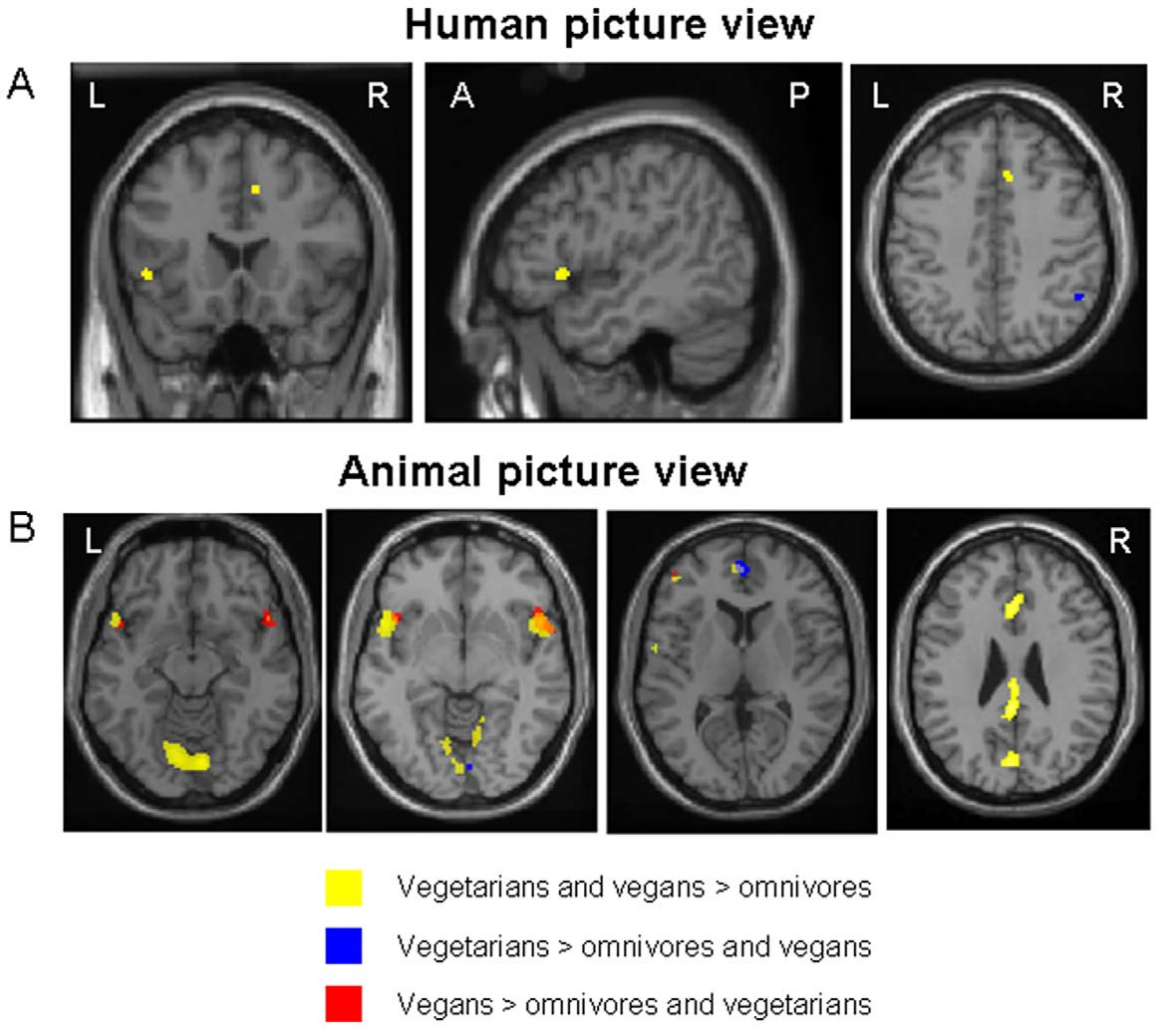
Results of the between-group comparisons of emotional (human and animal) negative valence picture views. Results are superimposed on a high resolution T1-weighted image in the standard MNI space, at a threshold of p<0.05 corrected for multiple comparisons. Areas activated during human picture view in vegetarians and vegans *vs.* omnivores are shown in yellow. Activations specific for vegetarians are shown in blue. Activations specific for vegans are shown in red. A: human picture view; B: animal picture view. Images are in neurological convention.

During human negative valence picture view, omnivore subjects had a more significant activation (p<0.05, FWE) of the bilateral middle temporal gyrus (MTG) (MNI space coordinates: 38, −58, 8, t value = 5.65; and −36, −76, 8, t value = 5.56) when compared to vegetarians and vegans. Compared to omnivore subjects, the entire sample of vegetarians and vegans had more significant activations (p<0.05, FWE) of the ACC (MNI space coordinates: 10, 22, 40; 10, 36, 28, and −4, 30, 36; t values = 5.65, 5.43, and 5.30), and the left inferior frontal gyrus (IFG) (MNI space coordinates: −48, 20, 0, t value = 5.56) (Figure 3).

During animal negative valence picture view, omnivore subjects had more significant activations (p<0.05, FWE) of the bilateral MTG (MNI space coordinates: −46, −62, 0, t value = 6.03; and 34, −74, 4, t value = 5.94), when compared to vegetarians and vegans. Compared to omnivore subjects, the entire sample of vegetarians and vegans had more significant activations (p<0.05, FWE) of the bilateral IFG (MNI space coordinates: −50, 14, −2, t value = 6.84; and 52, 14, −4, t value = 6.34), bilateral lingual gyrus (MNI space coordinates: 8, −80, −14, t value = 6.83; and −10, −78, −14, t value = 6.58), ACC (MNI space coordinates: 0, 24, 28; −2, 52, 8; t values = 5.76 and 5.51), posterior cingulate cortex (PCC) (MNI space coordinates: 0, −42, 26, t value = 5.87), left cuneus (MNI space coordinates: −2, −78, 24, t value = 5.83), and left middle frontal gyrus (MFG) (MNI space: −44, 46, 8, t value = 5.50) (Figure 3). This analysis also showed that, compared to omnivores, vegetarians and vegans had a lower activation of the right amygdala (MNI space coordinates: 30, 2, −20, t value = 5.38). To better define amygdala behavior in the three groups of subjects, we analyzed its activations and deactivations during the two experimental conditions in each group (Tables 1 and 2). This analysis revealed no significant activation neither deactivation (even when lowering the threshold for the statistical significance at a p<0.001, uncorrected) during animal picture view in this region in vegetarians and vegans.

**Table 2:** Cluster maxima coordinates of activations/deactivations, at the within-group one sample t test analysis of the areas which showed a significant interaction between groups and conditions (p<0.001, uncorrected).

	Human *vs*. neutral pictures			Animal *vs*. neutral pictures		
	Omnivores	Vegetarians	Vegans	Omnivores	Vegetarians	Vegans
Activation sites	MNI coordinatesX Y Z	MNI coordinatesX Y Z	MNI coordinatesX Y Z	MNI coordinatesX Y Z	MNI coordinatesX Y Z	MNI coordinatesX Y Z
R amygdala	32, 2, −24	28, −14, −16	30, −8, 22	26, 0, −24	-	-
L amygdala	−22, −4, −16	−24, −12, −18	−24, −6, −24	−22, −4, −20	-	−22, −2, −24
ACC	12, 36, 22	−4, 34, 26	−16, 46, −8	6, 36, 20	14, 46, −12	−12, 24, 28
R IFG	50, 30, 14	50, 28, 8	50, 30, 2	50, 32, 14	44, 16, 20	48, 28, −2

MNI = Montreal Neurological Institute, R = right, L = left, IFG = inferior frontal gyrus, ACC = anterior cingulate cortex.

Note that none of the regions shown in the table was significantly deactivated (one-sample t test).

### Different regions of activations between vegetarians and vegans

We also directly compared the neural responses in empathy and emotion-related networks between omnivores, vegetarians, and vegans, using a masking procedure (See Methods), to identify regions of specific activations of each group contrasted to the others.

#### a) Vegetarians vs. omnivores and vegans

Observation of human negative valence scenes resulted in a selective recruitment of the right IPL (BA40) (MNI space coordinates: 52, −50, 40, t value = 4.44) in vegetarians (Figure 3). For animal pictures, activations specific to vegetarians were found in the ACC (MNI space coordinates: −2, 52, 10, t value = 5.02) and the right lingual gyrus (MNI space coordinates: 8, −84, −10, t value = 5.00) (p<0.05, FWE).

#### b) Vegans vs. omnivores and vegetarians

During human negative valence picture view, no cortical activation “specific” to vegans was found. During animal negative valence picture view, vegans activated the IFG bilaterally (MNI space coordinates: 54, 16, −6, and −46, 18, −2, t values = 4.88 and 4.67), and the left MFG (BA10) (MNI space coordinates: −46, 48, 4, t value = 4.29) (Figure 3) (p<0.05, FWE).

### Analysis of interaction

**Figure 4:**
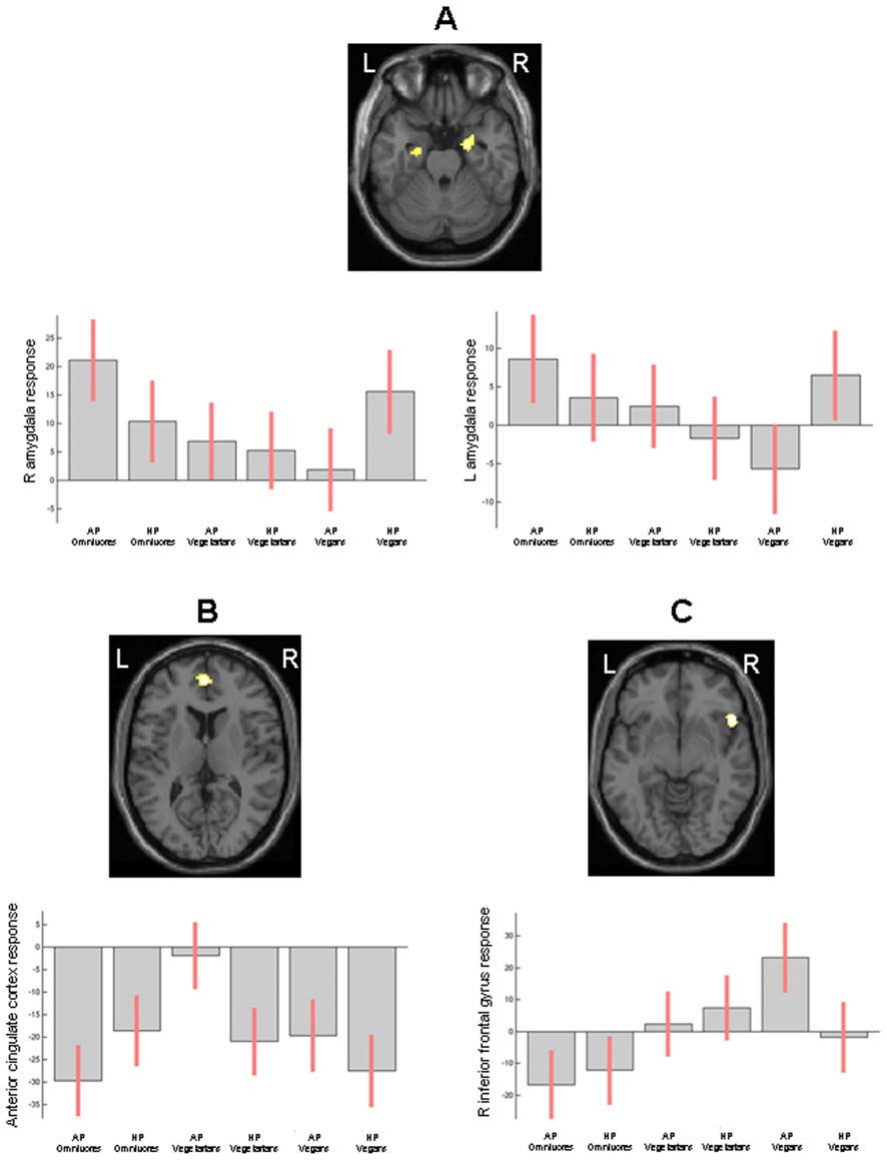
Interactions between stimuli (animal/human) and groups (omnivore/vegetarian/vegan). An interaction was found in the right amygdala (A), indicating greater increase to animal negative valence picture view in omnivores and to human negative valence picture view in vegans. An interaction between “human pictures” and “vegan group” was also found in the left amygdala (A). An interaction was found in ACC (B) between the “omnivore group” and “human pictures”, as well as between “vegetarian group” and “animal pictures”; and in the right IFG between “animal pictures” and “vegan group” (C). Foci of activations are shown on a high-resolution T1-weighted image in the standard MNI space. Plots indicate activation changes detected in the three groups during the two experimental conditions in each of these regions. Images are in neurological convention.

To further explore the specificity of stimulus processing within the three groups of subjects, we performed an analysis of interaction between picture types (animal/human) and groups (omnivore/vegetarian/vegan). Results showed an interaction in the right amygdala (MNI space coordinates: 24, −10, −22) (greater increases to animal negative valence view in omnivores and to human negative valence view in vegans) (Figure 3), the left amygdala (−22, −8, −28) (greater increases to human negative valence view in vegans) (Figure 4), the ACC (MNI space coordinates: −2, 52, 10) (preferential increases to human negative valence view in omnivores, and to animal negative valence view in vegetarians) (Figure 4); and the right IFG (MNI space coordinates: 52, 20, −8) (selective responses to animal negative valence view in vegans) (Figure 4).

Table 2 summarizes the behavior, in terms of activations/deactivations, at the within-group one sample t test analysis of the three main areas which showed a significant interaction between groups and conditions (i.e., amygdala, IFG, and ACC).

### Analysis of correlations

During human negative valence picture view, no correlation was found between EQ score and fMRI activity in the three groups of subjects of the study.

During animal negative picture view, significant correlations (p<0.001) were found between EQ score and:

- activation of the left MTG (r = 0.87), ACC (r = −0.76) and the bilateral IFG (right IFG: r = −0.71, left IFG: r = −0.89) in omnivores;
- activation of the left IFG (r = 0.92), the left MFG (r = 0.68), and the right MTG (r = −0.75) in vegetarians;
- activation of the bilateral lingual gyrus (right lingual gyrus: r = 0.69, left lingual gyrus: r = 0.75) and the left IFG (r = 0.78) in vegans.

## Discussion

The first main finding of this study was the demonstration of a common functional architecture of emotional processing in vegetarians and vegans. In particular, while omnivores are characterized by a greater activation of the bilateral posterior MTG during both human and animal negative valence scenes, vegetarians and vegans have constantly an higher engagement of empathy related areas while observing negative scenes, independently of the species of the individuals involved, which is characterized by an increased recruitment of the ACC and the IFG. Increased activation in the ACC and left IFG in vegetarians and vegans during human and animal suffering view is likely to reflect a stronger empathic response in the first two groups.

Remarkably, vegetarians and vegans have an higher engagement of empathy related areas while observing negative scenes regarding animals rather than humans, with the additional recruitment of the mPFC, PCC, and some visual areas. ACC has been associated with alert states, self awareness and pain processing [12], whereas mPFC and PCC activations are frequently observed in conditions involving representation of the self and self values [13]. The PCC is also thought to be involved in memory and visuospatial processing [14], particularly in relation to emotions and social behavior [13]. PCC is consistently activated when subjects have to judge the valence of emotionally salient words or episodic memories, with the strongest responses seen when unpleasant stimuli are presented [14].

The notion that empathic response might differ among vegetarians, vegans and omnivores, and that such a response might vary during viewing of human and animal sufferance is at least partially supported by the results of EQ assessment in the three groups of subjects and by the analysis of correlation between EQ scores and fMRI findings, which showed a direct relationship between the EQ score and left IFG recruitment during animal suffering view in vegetarians and vegans, whereas in omnivores such a relationship was inverse.

The pattern of increased recruitment of empathy-related areas in vegetarians and vegans during animal suffering view was also associated with a reduced activation of the right amygdala in comparison to omnivores. The amygdala responds to various kinds of aversive stimuli, most strongly fearful and threatening scenes [15] and, to a lesser extent, to those associated with disgust [16]. Remarkably, the within-group analysis during animal picture view, showed the absence of signal changes (in terms of activations and deactivations) within the amygdala in vegetarians and vegans, suggesting a down-regulation of amygdala response from areas located in the frontal lobes, in an attempt to regulate emotion through cortical processes in these subjects.

The second main finding of this study is the demonstration of strong functional architecture differences between the vegetarians and vegans during observation of negative scenes. During human suffering viewing, activations specific to vegetarians were located along the IPL. The IPL is involved in bodily representations that distinguish the self from the other [3], and was found to be more activated when pictures of mutilations were presented than when contamination or neutral pictures were shown[17], which suggests a stronger effect on the somatosensory system in observers exposed to the former than the latter conditions.

More critically, for animal pictures, activations specific to vegetarians were found in the ACC and the lingual gyrus, whereas activations specific to vegans were found in the bilateral IFG and the left MFG. Our data, therefore, point to differential ACC responses to animal suffering for vegetarians, a region highly interconnected with limbic and prefrontal structures that is thought to play a key role in normal and dysfunctional emotional self-control as well as social behaviour [18]. ACC activation has been related to awareness of emotional material, attention to emotional stimuli [19], and rating of affect intensity. In a meta-analysis study, Phan et al. [20] found that emotional tasks with explicit cognitive components (e.g., recognition or evaluation of emotional stimuli and biographic material) engaged specifically the ACC as compared to passive emotional conditions. The ACC has also been associated with alertness and attention, notably in terms of response control and during painful stimulation [21]. The recruitment of this region in vegetarians might therefore correspond to their distinctive behavioral response to pictures of animal suffering, e.g., enhanced attention and empathic pain [21], or increased self control and monitoring [22]. On the other hand, the activation of the inferior prefrontal cortex (IFG) seen in vegans during animal suffering, which is consistent with a role of such a region in different emotional tasks [20], may be related to aspects of cognitive control during emotion processing. Notably, right IFG is critically involved in inhibitory processes during both cognitive [23] and emotional [24] conditions. In addition, even if the existence of the mirror-neuron system (MNS) in humans is still controversial, the IFG is also considered to be part of such a system, since these regions are often activated during action observation, motor learning and imitation of action [25]. Activation of MNS areas has been shown to increase during social interaction, as well as during observation and imitation of emotional faces [25]. The role of the MNS in social cognition is also supported by studies in patients with autism, who show a reduced recruitment of the MNS, and in particular of the IFG, during observation and imitation of facial expressions [25]. Our findings therefore suggest a distinctive pattern of empathic response and emotional control in vegans, mediated through the IFG and MFG.

Between-group differences in stimuli processing were also confirmed by an analysis of interaction, which showed greater increases to animal negative valence view in omnivores and to human negative valence view in vegans in the amygdala, a preferential increase to human negative valence view in omnivores, and to animal negative valence view in vegetarians in the ACC, and selective responses to animal negative valence view in vegans in the right IFG. Intriguingly, an inverse correlation between amygdala response and activation in the right PFC and ACC has previously been shown during emotional tasks [26]. In humans, this system is thought to control and direct emotional responses through appraisal and evaluation of their experiences. Such an inverse correlation (i.e., decreased activation of the amygdala together with increased activations of the ACC and PFC) has also been demonstrated during “reappraisal”, which implies altering the meaning of a potentially emotion-eliciting situations in order to reduce their emotional impact [27], suggesting that cortical networks of prefrontal regions can exert a cognitive modulation on emotion processing in the amygdala, particularly during intense emotional responses. An alternative hypothesis that has been considered is that limbic structures, such as the amygdala, might respond preferentially to emotive stimuli at a sensory level, and less likely to be engaged in the cognitive processing of emotional material [28].

Collectively, our results reveal that distinct brain responses are evoked by emotionally significant pictures of humans and animals in people with vegetarian and vegan feeding habits, as well as between vegetarians and vegans, suggesting that different motivational factors might underlie their preferences and moral attitudes. Vegetarians showed distinctive responses to negative valence scenes of animals in the ACC, but also to negative valence scenes of humans in the IPL, which might be consistent with greater empathic pain responses and/or enhanced attention in this group for these two conditions. On the other hand, the selective response of vegans to animals in the ACC (with reduced amygdala responses) might reflect a greater attribution of self-relevance [13] and a greater recruitment of emotional regulation mechanisms [15], [26] when viewing negative states of non-human beings, together with an enhanced activation of the motor MNS and inhibitory control processes mediated through the MFG and the IFG, respectively. By contrast, omnivores, showed greater responses to human negative valence scenes in the ACC (together with reduced amygdala activation), suggesting that self-relevance and emotion control mechanisms were more specifically engaged by viewing suffering conspecifics than suffering animal beings.

Our study is the first to assess the neural correlates of empathy towards non conspecifics in people with different social norms, as reflected by their feeding habits. Our results converge with theories that consider empathy as accommodating a shared representation of emotions and sensations between individuals, allowing us to understand others [3]. They also led us to speculate that the neuronal bases of empathy involve several distinct components including mirroring mechanisms [25], as well as emotion contagion and representations of connectedness with the self [29]. In addition, brain areas similar to those showing different emotional responses between groups in our study (such as the IFG and the mPFC) have also been found to be modulated by religiosity [30], further supporting a key role of affect and empathy in moral reasoning and social values.

This study is not without limitations. First, the use of neutral scenes as a “baseline” condition does not allow defining the neural response to suffering per se, since the response might be influenced by seeing humans or animals. Second, even if a questionnaire related to feeding habits and the EQ were obtained from all the study subjects, affective and cognitive responses during fMRI acquisition were not recorded. Clearly, further studies are warranted to confirm our results.

## Materials and Methods

The study was approved by the Ethics Committee of Scientific Institute and University Ospedale San Raffaele, Milan, Italy and a written informed consent was obtained from all subjects prior to study entry, according to the Declaration of Helsinki.

### a) Subjects

We studied 60 right-handed [31] healthy subjects (34 women, and 26 men, mean age = 37.7 years, range = 18–60 years), with different dietary habits. All subjects had normal or corrected-to-normal vision. We recruited 20 omnivore subjects (11 women and 9 men; mean age = 36.9 years, range = 22–60 years), 19 vegetarians (11 women and 8 men; mean age = 40.3 years, range = 23–60 years), and 21 vegans (12 women and 9 men; mean age = 36.3 years, range = 18–53 years). The groups did not statistically differ for sex and age. A questionnaire was filled in by all the subjects before fMRI acquisition to investigate feeding habits, reasons/motivations of the feeding choices, and the time elapsed from such a choice. All vegetarians and vegans reported to have made their feeding choice for ethical reasons. They had stable feeding habit since 3.8 years (SD = 8.7 years), and were recruited among vegetarian associations. Omnivore subjects were recruited by advertisement and none of them had been vegetarian or vegan before the study. Eight vegans had been vegetarians before becoming vegans. All the subjects were naïve about the goal of the study. None of the subjects had any history of neurological, major medical, or psychiatric disorders (including depression), and either alcohol or drug abuse. In addition, none of the subjects was taking any medical treatment at the time of fMRI assessment and all of them had a normal neurological examination.

### b) Empathy assessment

On the day of fMRI acquisition, subjects were evaluated with the EQ questionnaire [32], a self-report questionnaire which has been developed to measure the cognitive and affective aspects of empathy. This questionnaire is widely used in clinical research [32], [33], as well as in neuroscience studies [34]. The EQ comprises 60 questions: 40 questions tapping empathy, and 20 filler/control items. The 20 filler/control items have been included to distract the participant from a relentless focus on empathy. On each empathy item, a person can score 2, 1, or 0, so that the EQ has a maximum score of 80 and a minimum score of 0. To avoid response bias, approximately half of the employed items are worded to produce a “disagree” response and half to produce an “agree” response [32]. The EQ has a forced choice format, can be self-administered, and is straightforward to score because it does not depend on any interpretation.

### c) Experimental design

During fMRI, an event-related design was used. A program implemented with the Presentation software (www.neuro-bs.com, Version 9.70) presented in a random order a series of 150 pictures: 40 showed negative valence scenes related to humans, 40 negative valence scenes related to animals, and the remaining 70 showed “neutral” natural landscapes. Pictures were pseudo-randomized so that no more than two pictures of the same category were presented consecutively. Negative-valence scenes were taken from the International Affective Picture System [35], newspapers, books, or magazines (all images were of high-quality resolution and taken in an electronic format). Scenes had to show the entire figure and not only the face of the subject/animal. Human and animal pictures were comparable in terms of valence and arousal rating. Non-IAPS pictures were validated in a group of 50 healthy subjects that did not participate in the fMRI experiment. To assess the three dimensions of pleasure, arousal, and dominance, the rating procedure by Lang was used [35].

Each trial began with a fixation cross presented in the centre of the screen for 3 sec, followed by the pictures, in a random order, presented for 2 sec followed by black screen. A variable interstimuls interval was used. Subjects were instructed to look at the scenes, without providing any specific response during fMRI acquisition.

### d) fMRI acquisition

Brain MRI scans were obtained using a 3.0 Tesla scanner (Intera Philips Medical Systems, Best, The Netherlands) with a gradient strength of 40 mT/m. Functional MR images were acquired using a T2*-weighted single-shot echo-planar imaging (EPI) sequence (echo time [TE] = 30 ms, flip angle = 85°, matrix size = 128×128, field of view [FOV] = 240 mm^2^, repetition time [TR] = 3.0 seconds). During each functional scanning run, 151 sets of 40 axial slices, parallel to the AC-PC plane, with a thickness of 3 mm, covering the whole brain were acquired. Shimming was performed for the entire brain using an auto-shim routine, which yielded satisfactory magnetic field homogeneity. Head movements were minimized using foam paddings.

On the same occasion, a brain dual-echo turbo spin echo sequence (TR = 3500 ms, TE = 24/120 ms; echo train length = 5; flip angle = 150°, 44 contiguous, 3-mm-thick, axial slices with a matrix size = 256×256 and a FOV = 240×240 mm^2^) was also acquired.

### f) FMRI analysis

FMRI data were analyzed using the statistical parametric mapping (SPM2) software. Prior to statistical analysis, all images were realigned to the first one to correct for subject motion, spatially normalized into the Montreal Neurological Institute (MNI) space, and smoothed with a 10-mm, 3D-Gaussian FWHM filter.

### g) Statistical analysis

Event-related paradigms for each condition were modelled on a voxel-by-voxel basis, using the general linear model and the theory of random Gaussian fields [36]. In each subject, a first-level design matrix was built, where motion parameters were used as regressors of no interest. Then, specific effects were tested by applying appropriate linear contrasts. For each subject, the following contrasts were defined: human negative valence images > neutral, and animal negative valence images > neutral. To test whether between-group differences in processing the neutral conditions might have influenced our results, the contrast assessing activations of neutral images was also defined. Significant hemodynamic changes for each contrast were assessed using t statistical parametric maps (SPMt). Then, a second level random effect analysis was performed to assess the main effects of the stimuli, differences between groups, and interactions between groups and conditions [37], using an ANOVA model where groups and conditions were entered as separate factors (2×3 factorial design). To assess between-group similarities and differences in the brain patterns of activations, the following sets of linear comparisons were performed: 1) vegetarians and vegans, separately, *vs.* omnivores; 2) vegetarians and vegans, combined, *vs.* omnivores; 3) vegetarians *vs.* vegans, and vice versa. Common patterns of activations between vegetarians and vegans during a given contrast were identified by a conjunction analysis [38]. Regions of specific activations of each group contrasted to the other were identified by inclusively masking (uncorrected mask p value = 0.05) the relevant contrast from comparison 1 (e.g., vegetarians *vs.* omnivores) with the appropriate contrast from comparison 3 (e.g., vegetarians *vs.* vegans).

Intra-group activations were evaluated using a one-sample t test and a paired t test, as appropriate. At this stage, task-related activations and deactivations were estimated. We report activations below a threshold of p<0.05 corrected for multiple comparisons (FWE). Within each region of statistical significance, local maxima of signal increase were determined and their locations expressed in terms of *x*, *y*, and *z* coordinates into the MNI space. A 3D anatomical atlas was also used to increase confidence in the identification of the anatomical locations of the activated areas [39]. Using a linear regression analysis, the correlation of fMRI changes during task performance with EQ score was assessed (p<0.001, uncorrected).

Demographic and behavioral data were compared using the SPSS software and an ANOVA model (version 13.0).
